# Analysis of temperature dependence of background phase errors in phase-contrast cardiovascular magnetic resonance

**DOI:** 10.1186/s12968-014-0097-6

**Published:** 2014-12-11

**Authors:** Julia Busch, S Johanna Vannesjo, Christoph Barmet, Klaas P Pruessmann, Sebastian Kozerke

**Affiliations:** Institute for Biomedical Engineering, University of Zurich and ETH Zurich, Zurich, Switzerland; Skope Magnetic Resonance Technologies, Zurich, Switzerland; Division of Imaging Science and Biomedical Engineering, King’s College London, London, UK

**Keywords:** Cardiovascular magnetic resonance, Phase-contrast, 4D flow, Background phase error, Thermal stability, Magnetic field monitoring

## Abstract

**Background:**

The accuracy of phase-contrast cardiovascular magnetic resonance (PC-CMR) can be compromised by background phase errors. It is the objective of the present work to provide an analysis of the temperature dependence of background phase errors in PC-CMR by means of gradient mount temperature sensing and magnetic field monitoring.

**Methods:**

Background phase errors were measured for various temperatures of the gradient mount using magnetic field monitoring and validated in a static phantom. The effect of thermal changes during k-space acquisition was simulated and confirmed with measurements in a stationary phantom.

**Results:**

The temperature of the gradient mount was found to increase by 20–30 K during PC-CMR measurements of 6–12 min duration. Associated changes in background phase errors of up to 11% or 0.35 radian were measured at 10 cm from the magnet’s iso-center as a result of first order offsets. Zeroth order phase errors exhibited little thermal dependence.

**Conclusions:**

It is concluded that changes in gradient mount temperature significantly modify background phase errors during PC-CMR with high gradient duty cycle. Since temperature increases significantly during the first minutes of scanning the results presented are also of relevance for single-slice or multi-slice PC-CMR scans. The findings prompt for further studies to investigate advanced correction methods taking into account gradient temperature and/or the use of concurrent field-monitoring to map gradient-induced fields throughout the scan.

## Background

Phase-contrast (PC) cardiovascular magnetic resonance (CMR) is able to provide time-resolved velocity data of blood flow in a single or multiple slices as well as with volumetric coverage (4D PC-CMR) [1]. The velocity information in the data allows for the calculation of hemodynamic parameters critical in the assessment of cardiac pathologies such as coarctation and stenosis [2-5]. Volume flow through cross-sections of the main arteries and veins is a key parameter to measure cardiac output, shunt flow and regurgitation [3]. While these parameters are typically extracted from through-plane velocity encoded single-slice PC-CMR measurements in a clinical setting, more recent work indicates the advantage of acquiring 4D PC-CMR data to enable retrospective analysis of flow parameters in a number of defined planes e.g. through the aortic and mitral valves [6,7]. Furthermore, streamline and pathline visualization [8,9] offer an intuitive understanding of the complex flow patterns.

In general, PC-CMR may be compromised by background phase errors induced by the velocity encoding gradients. Phase errors are caused by concomitant gradient fields, gradient non-uniformity and eddy-current effects. The term gradient non-uniformity herein refers to the deviation from nominal gradient strength and orientation.

To a first approximation, concomitant gradient fields cause 2^nd^ order spatial field offsets which can be calculated analytically and corrected for in post-processing [10,11]. Gradient non-uniformity leads to geometric distortions and deviation in encoding velocity and direction. The effects can be corrected for using a generalized reconstruction taking into account a theoretical model of gradient non-uniformity [12] or scaling factors acquired in phantom measurements [13]. Eddy-current induced phase errors are mainly exponential in time and predominantly of 0^th^ and 1^st^ spatial order. On most clinical CMR scanners they are compensated for using hard- or software gradient pre-emphasis [14].

Despite all these successful correction approaches phase errors remain. Residual errors were found to be primarily caused by oscillatory field fluctuations due to mechanical resonances of the gradient coils [15].

Correction of background phase errors is important to ensure accuracy of derived parameters used for clinical diagnostics. Upon performance of a multi-centre multi-vendor study to investigate background phase errors, Gatehouse et al. [16] stated a limit of acceptability of 5% error in stroke volume or, equivalently, 0.4% of the encoding velocity. The study also showed that for such an accuracy to be achieved post-processing is required on all systems [16]. Besides effects on hemodynamic parameters, offset errors also compromise the accuracy of flow visualization causing streamlines and particle tracks to show non-physiological behaviour by passing through vessel walls [17].

For two-dimensional PC-CMR with one-directional flow encoding two background phase correction approaches have been proposed: a) repetition of the phase contrast sequence on a stationary phantom and b) referencing in stationary tissue [18,19]. Data acquisition on a stationary phantom provides a map of the phase error over the field of view (FOV) which can be subtracted from the data of interest. For referencing in stationary tissue the background offsets are typically assumed to be linear over the image [20]. Stationary tissue is identified and the offset estimated by fitting a linear function through the reference tissue. While the additional phantom scan is more accurate if the background error is nonlinear, it adds to the overall complexity and time of data acquisition. Referencing in stationary tissue, on the other hand, requires sufficient stationary tissue and good SNR and data quality.

Today, correction of background phase errors by linear regression through stationary tissue or a separate phantom scan is common for both one-directional and three-directional flow encoding.

Similar to pre-emphasis for non-oscillating eddy currents, pre-emphasis compensation for oscillatory phase offsets has been described [21]. While this approach aims at preventing background phase errors, it has not been used routinely so far.

For these correction methods to work, some prerequisites have to be met: low spatial order of the background offsets in case of referencing in stationary tissue and temporal stability for a correction based on an additional phantom scan. Recently, it was shown that background phase offsets are reproducible within scanning sessions. However, long-term drifts over several months exceeding the limit of 5% error in stroke volume can occur which would prevent a correction using pre-stored parameters [22]. Furthermore, temporal stability during scanning with prolonged periods of high gradient duty cycle has been a concern. Gatehouse et al. [22] hypothesized that such instability is caused by thermal changes of the gradients. This may be of particular relevance if multi-directional flow encoding and new multi-venc approaches [18,23-25] with increased gradient load and long scan times are employed.

It is hypothesized here that the high gradient duty cycle required for PC-CMR result in significant temperature changes of the gradient mount thereby modifying mechanical eigenmodes and hence oscillatory phase offsets in PC-CMR. The term “gradient mount” refers to the supporting structure carrying the gradient current leads.

The objective of the present work is to provide an in-depth analysis of background phase errors in PC-CMR under thermal changes of the gradient mount by means of gradient mount temperature sensing and magnetic field monitoring.

## Methods

To analyse background phase errors under thermal changes, a two-dimensional slice with three-directional velocity encoding was acquired repeatedly within the same scan. To simulate the gradient load exhibited during a 4D PC-CMR measurement, data was acquired with corresponding timing. Data was acquired both with magnetic field monitoring [26] and in a stationary phantom. Additionally, gradient impulse response functions [27-29] were measured for all three gradient axes under various thermal conditions.

### Temperature measurement setup

**Figure 1:**
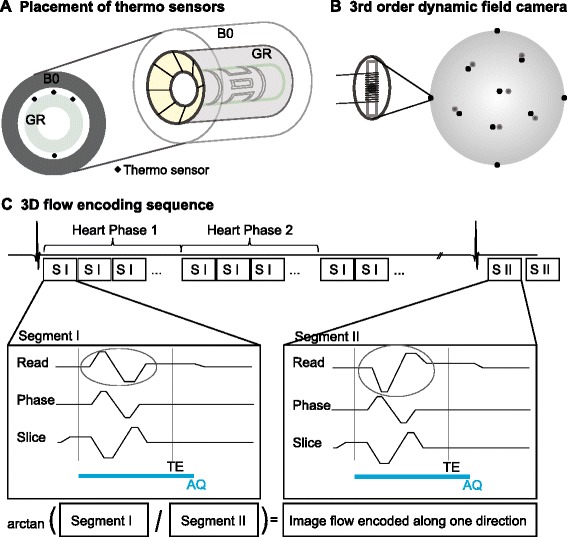
**Experimental setup.** Positioning of thermo-sensors on gradient mount **(A)**; schematics of third order dynamic field camera **(B)** and three-directional PC-CMR sequence used for flow encoding **(C)**. The acquisition window (AQ) during which field-monitoring data were acquired is indicated.

Four fibre-optic thermo sensors were installed on the gradient mount of a 3 T Philips Achieva System (Philips Healthcare, Best, The Netherlands) as demonstrated in Figure 1A. Using a Luxtron 790 fibre optic temperature measurement setup (LumaSense Technology, Santa Clara, CA, USA) the temperature was monitored and recorded during all scans.

### Sequence parameters

With an isotropic in-plane resolution of 1.75 mm a field of view (FOV) of 320×257 mm^2^ was covered. A partial-echo factor of 0.75 was applied; slice thickness was 10 mm and flip angle was 10^0^. Datasets were acquired using symmetric 4-point velocity encoding (Figure 1C) in non-angulated sagittal, transverse and coronal slices through the iso-center with isotropic encoding velocities (venc) of 50, 100 and 150 cm/s each. For sagittal and transverse slice orientation TE/TR was 1.85 ms/3.9 ms, 2.0 ms/4.1 ms and 2.5 ms/4.5 ms for a venc of 150, 100 and 50 cm/s, respectively. For coronal slice orientation TE/TR was 1.8 ms/3.9 ms, 2.0 ms/4.1 ms and 2.4 ms/4.5 ms for a venc of 150, 100 and 50 cm/s, respectively. The four velocity encoding segments were acquired beat-interleaved i.e. segments were alternated in intervals of the simulated heartbeat. Upon initial tests it was confirmed that the temperature increases exponentially reaching a steady level after 10 to 15 minutes of constant gradient activity. Hence, to cover the whole range of possible temperatures, the number of repetitions of the 2D slice acquisition was chosen accordingly and was set to 300 in case of venc 150 and 100 cm/s (total scan duration: 12 min) and 250 for venc 50 cm/s (total scan duration: 11 min).

All nine configurations (3 orientations +3 vencs each) were measured both with magnetic field monitoring and on a stationary phantom.

### Magnetic field monitoring

For magnetic field monitoring a third order dynamic field camera [30] (Skope Magnetic Resonance Technologies, Zurich, Switzerland) consisting of 16 NMR probes arranged on a 20 cm diameter sphere was used (Figure 1B). The acquisition window was adjusted to cover almost the full repetition time from the centre of the slice-select gradient to the end of the read-out gradient (Figure 1C). Phase differences were calculated up to 2^nd^ spatial order [26]. Longitudinal and transversal relaxation time constants of the field probes were 105 ms and 100 ms. Thus, repeated excitation with short repetition times as used in PC-CMR would have resulted in unwanted saturation and echoes corrupting the field probe signal. To solve this, the spacing between successive excitations was artificially prolonged: gradient pattern, timing and activity were maintained the same as in the phantom measurements, however, only the central phase-encode line was excited and acquired. This is feasible since only phase differences were required and hence effects from slice-select, phase-encode and read-out gradients are cancelled. Initial tests confirmed this assumption. The reduction in the number of RF excitations and the prolonged time between successive excitations guaranteed good quality and high SNR of the field monitoring data.

### Phantom measurements

Phantom measurements were performed on a stationary 20 cm diameter spherical phantom using an 8-element head coil receive array. To avoid motion artefacts due to movement of the phantom fluid during measurements water was gelled with agarose. The gel was doped with 0.13 mmol/l of Gadovist to obtain a longitudinal relaxation time on the order of 800 ms.

### Computer simulations

The effect of thermal changes of the gradient mount on phase errors in 4D PC-CMR was simulated. The 0^th^ and 1^st^ order phase at the echo time was extracted from monitoring data acquired in the transverse slice with venc 100 cm/s. The thermal change of these phase coefficients was then fitted to an exponential function to describe the phase at arbitrary time points, hence for the acquisition time of any k-space profile. Thirteen slices were simulated with a spatial resolution of 1.75×1.75×2 mm^3^ covering a FOV of 320×257×26 mm^3^. With a TFE factor of 12 (12 phase encode lines acquired per time frame), a simulated heartbeat of 1 s and beat-interleaved acquisition; this resulted in a simulated scan time of 14 min.

According to the Fourier Shift Theorem a linear phase in position space results in a shift in k-space. If the linear phase alters during data acquisition, each readout-line has a slightly different shift. In the simulation, background phase errors were compared using temperature dependent shifts versus shifting the full k-space according to the offset in the k-space centre.

To confirm the simulation, data was acquired in a stationary phantom using a 4D PC-CMR sequence with coronal orientation. The measured data was compared to simulations of the background phase errors using phase offsets at the k-space centre only which were measured with magnetic field monitoring. The sequence parameters were as follows; spatial resolution: 2×2×2 mm^3^, FOV: 320×256×10 mm^3^, TE/TR: 2.1 ms/4.2 ms, retrospective triggering, 18 heart phases, TFE factor: 9, venc: 150 cm/s, scan duration: 6 min (reduced scan time due to data size limitations on the scanner).

Before analysis, all phantom data was corrected for concomitant fields using the manufacturer’s image reconstruction software. The vendor’s post-processing filters for eddy-current correction were switched off. Data acquired with field monitoring were corrected for concomitant fields using the approach described in [11]. All data was analysed in Matlab (MathWorks, Natick, Massachusetts, USA).

## Results

### Thermal effects

**Figure 2:**
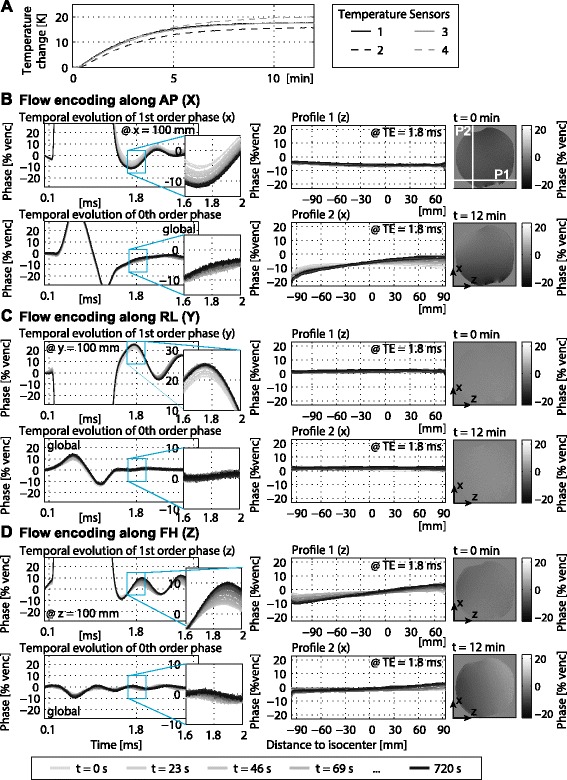
**Temperature increase and changes in phase errors in a sagittal slice.** Temperature increase recorded during a 12 min PC-CMR scan (venc: 150 cm/s, TE: 1.85 ms) **(A)**. Linear and zeroth order background phase errors measured at different temperatures for the x **(B)**, y **(C)** and z **(D)** gradient coils (left). Phase profiles in a stationary phantom acquired with the same sequence at the beginning and end of the scan (right).

Figure 2 shows an analysis of the thermal effects due to constant high gradient load for an exemplary dataset acquired with sagittal orientation and an encoding velocity of 150 cm/s. The temperature increase was 20 K over 12 minutes (Figure 2A).

Since the slice orientation was chosen non-angulated, patient coordinates align with the physical axes of the gradient fields. This implies that per reconstructed phase difference (e.g. antero-posterior (AP)) only one gradient coil has to be considered for analysis (e.g. G_x_). Thus, thermal effects can be separately analysed for the x, y and z gradient coils. Please note that on the 3 T Philips Achieva system the x-axis refers to the vertical axis while the y-axis refers to the horizontal axis; z denotes the axis parallel to B_0_.

The time evolution of the phase differences separated into 0^th^ order and the 1^st^ order self-term are shown in the left column for flow-encoding with the x (Figure 2B), y (Figure 2C) and z (Figure 2D) gradient coils. In the 1^st^ order self-term, the phase accumulated during the flow-encoding gradient is depicted. Due to mechanical gradient coil vibrations, phase differences are found to exhibit an oscillatory behaviour, which changes with increasing temperature.

The right column of Figure 2 depicts the background phase in the stationary phantom for the first and last scan repetition for flow encoding along the x, y and z gradient coils. Further, the phase along a horizontal and vertical profile through the iso-center is plotted (middle column). All data is scaled in percentage of the encoding velocity. First order phase differences are evaluated at a distance of 10 cm from the iso-center. The change from grey to black denotes an increase in scan repetition and hence an increase in temperature of the gradient mount. Please note that data were acquired in regular time steps and thus for exponentially saturating increase in temperature.

Considering phase differences for flow encoding along feet-head (FH) (Figure 2D) shifts in frequency and decay time of the oscillations can be observed. This results in a change in background phase error that depends on the echo time point in the sequence. The temperature dependent shifts in frequency and decay time become more prominent in the frequency domain analysis of the gradient impulse response functions (GIRFs) (see Appendix A1). For sagittal slice orientation with venc 150 cm/s the echo time is 1.85 ms. At this time point evaluated at 10 cm from the iso-center the background phase changes by up to 6.7 ± 0.3%venc for flow encoding along AP and up to 6.0 ± 0.3%venc for flow encoding along FH. The same thermal effects are reproduced in the phantom scan (middle and right column). For flow encoding along RL the background phase would change by up to 3.4 ± 0.3% at a distance of 0.1 m from the iso-center. However, RL denotes the through-plane direction and data was acquired in the iso-center, so this change is not visible in the phantom measurements.

Little change is observed with respect to non-oscillating eddy-currents. Both the phantom and the monitoring data demonstrate that with a temperature increase changes in the 0^th^ order are below 2.5%. 0^th^ order phase evolution shows a change in eddy-current behaviour which, however, cancels out before the echo time.

In the phantom data, some higher order phase errors can be observed. Since the higher orders show little to no temperature dependence, the 2^nd^ order monitoring data is not presented here. The change in background phase for all three axes for the dataset with sagittal orientation and venc 150 cm/s is summarized in Table 1.

**Table 1:** **Summary of change in phase offsets for sagittal slice orientation at flow-encoding velocity of 150 cm/s (TE =1.85 ms)**

	**1** ^**st**^ **order phase offset at 10 cm from iso-center [% venc]**			**0th order phase offset [% venc]**		
	**X → X**	**Y → Y**	**Z → Z**	**X → B0**	**Y → B0**	**Z → B0**
**T =0 min**	−2.1 ± 0.2	18.8 ± 0.2	3.1 ± 0.2	−6.8 ± 0.3	0.9 ± 0.3	−1.4 ± 0.3
**T =12 min**	−8.8 ± 0.2	22.2 ± 0.2	9.1 ± 0.2	−4.3 ± 0.3	0.9 ± 0.3	−0.3 ± 0.3
**Difference**	−6.7 ± 0.3	3.4 ± 0.3	6 ± 0.3	2.5 ± 0.4	0 ± 0.4	−1.1 ± 0.4

**Figure 3:**
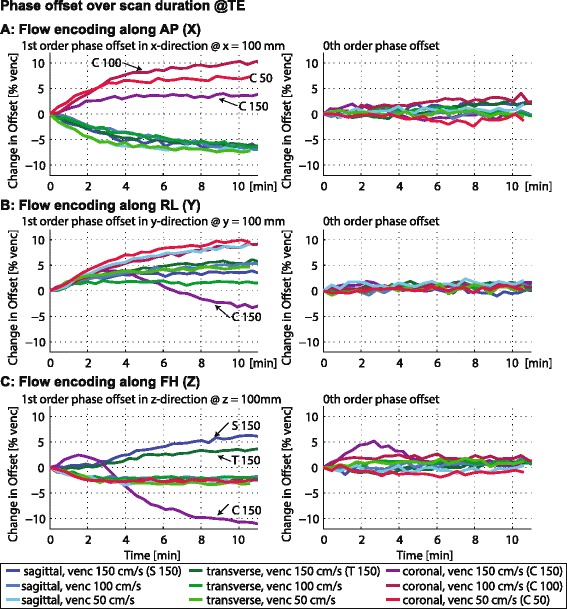
**Change in phase errors for different geometries and encoding velocities.** Linear and zeroth order background phase errors measured at different temperatures for the x, y and z gradient coils in sagittal, transverse and coronal slices with velocity encodings of 50, 100 and 150 cm/s. Velocity was encoded along AP **(A)**, RL **(B)** and FH **(C)**.

In Figure 3 the resultant change in background offset at the echo times extracted from the data acquired with field monitoring is summarized for all nine measurements. Changes in 1^st^ and 0^th^ order offsets are depicted for flow encoding along the x, y and z gradient coils. At 10 cm distance from the iso-center changes in phase error of up to 11% of the encoding velocity can occur over the duration of the scan (Figure 3C). Apart from coronal orientation with venc 150 cm/s changes in 0^th^ order offsets are below 2.5% of the encoding velocity. Accordingly, assuming standard phantom calibration at room temperature of the gradients vs. measurements at 20 K above room temperature, errors of more than 10% of the encoding velocity can occur in a 20 cm FOV over a measurement time of 12 minutes. Already after 4 minutes errors of up to 7.5% of the encoding velocity occur.

### Computer simulations

**Figure 4:**
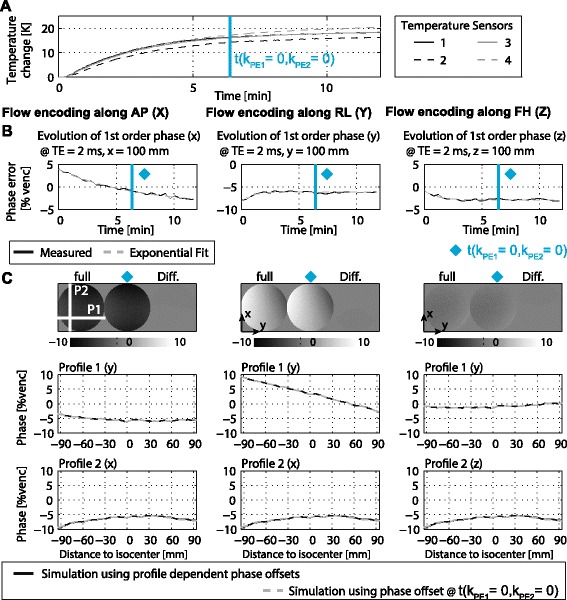
**Temperature dependency of phase errors in simulated 4D PC-CMR.** First order phase coefficients recorded during a 12 min scan generating a temperature increase of 20 K are used to calculate phase offsets in a numerical 4D PC-CMR phantom **(A-C)**. Comparison of simulations using temperature dependent phase offsets versus background phases calculated using only information about shifts at the k-space centre yield no difference **(C)**.

In Figure 4 simulation results based on measured temperatures (Figure 4A) and field monitoring data (Figure 4B) for a transverse scan with an encoding velocity of 100 cm/s are presented. Slices as well as profiles through a simulated spherical phantom (Figure 4C) show that simulations using temperature dependent phase offsets for each k-space profile are well approximated by the temperature dependent phase offset measured at the k-space centre.

**Figure 5:**
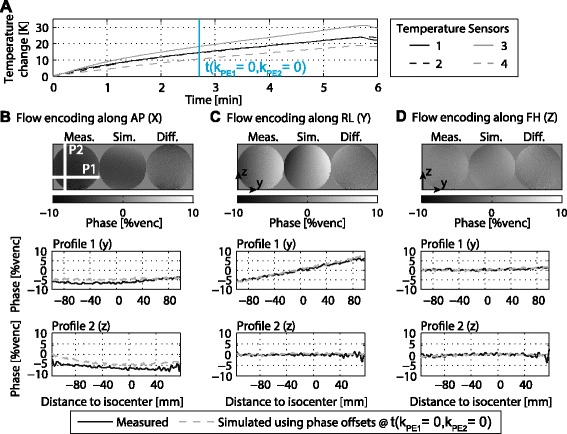
**Comparison of simulated and measured 4D PC-CMR sequence.** For 4D flow temperature changes by up to 30 K over the scan duration **(A)**. For flow encoding along AP **(B)**, RL **(C)** and FH **(D)** measured background phase errors are compared to simulations in which only information about errors of the central k-space line are used.

In Figure 5 simulations of background phase errors using phase offsets at the k-space centre are compared to phantom measurements acquired with the 4D PC-CMR sequence. During the measurement a temperature increase of 30 K was recorded (Figure 5A). The measured phase errors match the simulated phase errors in case of flow encoding along right-left (RL) and FH directions (Figures 5C,D). For flow encoding along AP small discrepancies are seen (Figure 5B).

## Discussion and conclusions

For multi-directional PC-CMR measurements with typical encoding velocities the temperature of the gradient mount was found to increase by up to 20–30 K for the MR system tested. GIRF measurements acquired at different temperatures of the gradient mount (A1) revealed that the frequency and decay times of oscillatory phase offsets decrease with increasing temperature. This observation suggests that the stiffness of the gradient mount is reduced at increasing temperatures. As a result, changes of the linear background phase offsets by up to 11% of the encoding velocity at 10 cm distance from the iso-center were recorded depending on the temperature of the gradient mount.

In first approximation, the changes in oscillation parameters result in temperature dependent shifts in k-space over the duration of the scan. Because of the slow change in k-space shifts (~11%venc/10 cm in 11 min) the corresponding linear phase ramps in image space are well captured by phase errors measured at the k-space centre only. The temperature of the gradient mount is crucial for the effective linear phase ramp while the k-space centre is sampled and thus the timing of the k-space centre within the sequence.

Analyses of GIRFs with respect to gradient channel delays revealed no thermal variation. This finding is in contrast to previous results by Brodsky et al. [31] who reported changes in gradient channel delays on the order of μs associated with changes in gradient coil temperature.

While simulated and phantom data agreed well for flow encoding along RL and FH directions, a mismatch between measured and simulated data was found for flow encoding along the AP direction. This discrepancy is associated with remaining 0^th^ order eddy currents and field oscillations from the previous TR at the point of excitation of the current TR. Due to the finite switching time of the transmit/receive switch of the scanner, monitoring data right after excitation could not be acquired and hence the phase difference right after excitation was assumed to be zero. This assumption, however, does not hold if decay times of eddy-currents are much longer than TR. In this case, the monitoring data exhibit an unknown offset. GIRF measurements of the x-gradient coil confirmed longer time constants both for 0^th^ order eddy-currents as well as for oscillatory field fluctuations when compared to the y- and z-gradients.

In case of 4D PC-CMR with coronal orientations (Figure 5) a temperature increase of 30 K was recorded over a scan duration of only 6 minutes. In contrast, a lower temperature increase by 20 K was measured during 12 minutes for 2D PC-CMR with sagittal orientation (Figure 2). This can be explained by the location of the thermo sensors and differences in gradient activity between the two scans. The thermo sensors are most sensitive to temperature changes caused by heating of the x-gradient coil. For isotropic velocity encoding the overall time of gradient activity (amplitude, duration and gradient switching) is highest along the read-out direction and lowest along the phase-encode direction. In case of coronal orientation, the read-out direction was chosen along the AP direction (x) while for sagittal orientation the read-out was chosen along the FH direction (z).

The change in temperature during k-space acquisition implies that flow data acquired with the same sequence parameters but different profile orders (linear vs. high-low vs. low-high) will have different background errors. Also, repetition of the exact same sequence can lead to different phase offsets if thermal conditions are different, e.g. a scan started with gradients at room temperature versus a scan started when the gradient mount has already been heated by previous measurements.

 While the data presented in this work aimed at analysing changes in background phase errors in 4D PC-CMR measurements, results may also be of relevance for single-slice or multi-slice 2D PC-CMR with similar gradient activity per unit time. Changes in background phase errors of up to 2.8% of venc were measured during the first minute of scanning suggesting even an impact on single-slice PC-CMR protocols employing multiple signal averages for respiratory motion compensation.

The extent of temperature related changes in background phase errors was found to depend on the echo time point (see Appendix A2). If the echo time point coincides with an extremum of the oscillatory fields, background phase offsets are largest and strongly depend on temperature. In contrast, if the echo time point is at an inflection point, background phase offsets show only little temperature dependency. This point may explain in parts the inconclusive findings of the work by Rolf et al. [32].

A shift in resonance frequency and a decrease in decay time of temporal field oscillations will render pre-emphasis compensation with fixed parameters problematic. At the minimum, pre-emphasis compensation would have to be adjusted to match thermal conditions at the k-space centre.

The measurement of the background error in a separate phantom scan is in principle feasible if the sequence can be repeated with exactly the same parameters and timing and under the same thermal conditions. Matching initial thermal conditions could be achieved by additional fixed times of scanner inactivity in-between measurement sessions and scans. In a busy clinical workday, however, this is challenging. Further, the use of retrospective gating and breathing navigators in connection with patient specific heart rate and breathing variations will further complicate the acquisition of an exact scan replica on the phantom.

Although the analysis of temperature dependent background phase errors presented here was conducted on a single MR system, similar effects can be expected on the various systems used in the field. Significant background phase errors were found on MR machines from all major vendors and a dependence of errors on system temperature was indicated [16,22].

Whereas only little work on the stability of PC-CMR is available so far, stability measurements are frequently reported by the neuro research community [33,34]. For functional magnetic resonance imaging and diffusion weighted imaging, frequency drifts due to heating of the shim irons are a concern. Also effects of mechanical vibrations of the gradient coils have been studied [35,36].

From the present study it is concluded that changes in gradient mount temperature significantly modify background phase errors during typical PC-CMR scans. This finding prompts for further studies to investigate advanced correction methods taking into account gradient temperature and/or the use of concurrent field-monitoring [37-39] to map gradient-induced fields throughout the scan.

## Figures and Tables

**Figure 6:**
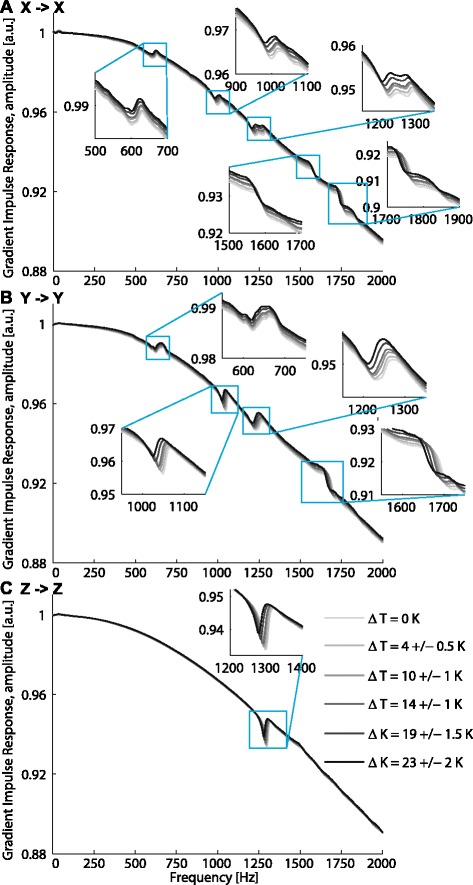
**Gradient-impulse response functions.** Self-term gradient-impulse response function for the x **(A)**, y **(B)** and z **(C)** gradient coil measured at different temperatures of the gradient mount.

**Figure 7:**
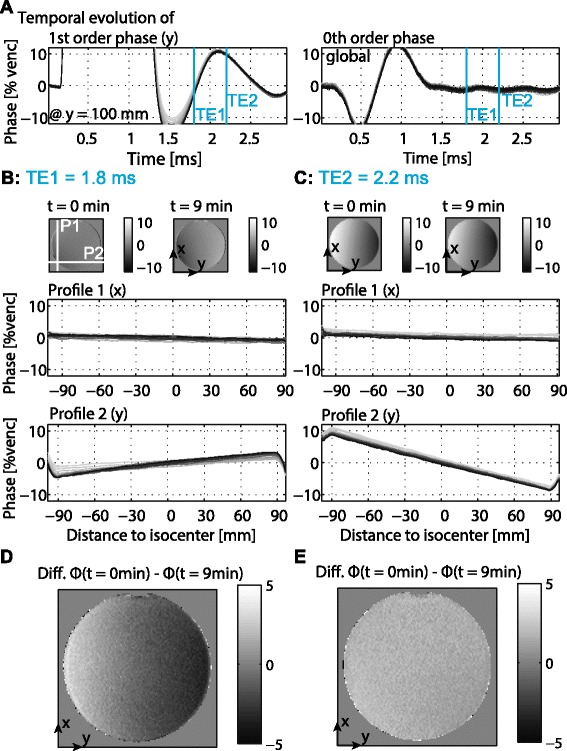
**Echo-time dependence of phase offsets.** Comparison of temperature dependent changes of first and zeroth order background phase errors **(A)** at two echo times (1.8 ms and 2.2 ms) **(B,C)**. Corresponding difference maps of phase images acquired at 0 and 9 min **(D,E)**.

## Appendix

### Appendix A1: Temperature-dependent GIRFs

Figure 6 shows the self-term gradient impulse response functions for the x, y and z gradient coils measured under various thermal conditions of the gradient mount. The scale from light grey to black indicates increasing temperatures.

In the frequency domain the damped oscillatory field fluctuations give rise to Lorentzian peaks, which are characterized by amplitude, frequency, phase and decay time.

The impulse response function of the z gradient coils exhibits one major peak at 1.3 kHz along some smaller and broader peaks between 1.4 and 1.8 kHz (Figure 6C). The impulse response for the x and y gradient coils shows a number of larger peaks ranging from 0.5 to 1.9 kHz (Figures 6A and B). Frequency and decay time decrease with increasing temperature. Variation of the gradient channel delays under thermal changes has not been observed in any experiment.

### Appendix A2: Echo time dependence

The amount of thermal fluctuation of the background phase error is dependent on the echo time point and can hence be reduced with a suitable choice of echo time. In Figure 7 the thermal changes are compared for an echo time point of 1.8 ms vs. 2.2 ms in a non-angulated transverse slice through the iso-center for flow encoding along RL (venc 150 cm/s). TR was extended to 4.5 ms to allow for prolonged echo times. For the shift in echo time only the read-out gradient was shifted, all other gradients were kept the same.

At an echo time of 1.8 ms the 1^st^ order phase difference is negligible at room temperature (B). After 9 minutes of scanning, however, it amounts to 5% venc. At an echo time of 2.2 ms a phase error of up to 10% venc (10 cm from iso-center) can be seen in the phase evolution and in the static phantom scan at room temperature (C). In this case the 1^st^ order phase remains unchanged even for higher temperatures of the gradient mount. Here a small change in 0^th^ order offset occurs. The difference in the thermal effects at echo time points of 1.8 and 2.2 ms are apparent in the difference plots shown in Figure 7D and E.
